# Use of Twitter data to improve Zika virus surveillance in the United States during the 2016 epidemic

**DOI:** 10.1186/s12889-019-7103-8

**Published:** 2019-06-14

**Authors:** Shahir Masri, Jianfeng Jia, Chen Li, Guofa Zhou, Ming-Chieh Lee, Guiyun Yan, Jun Wu

**Affiliations:** 1 Program in Public Health, College of Health Sciences, Uniersity of California, Irvine, California USA; 2 Department of Computer Science, University of California, Irvine, California USA

**Keywords:** Zika, ZIKV, Zika virus, Disease surveillance, Disease forecasting, Predictive modeling, Autoregressive model

## Abstract

**Background:**

Zika virus (ZIKV) is an emerging mosquito-borne arbovirus that can produce serious public health consequences. In 2016, ZIKV caused an epidemic in many countries around the world, including the United States. ZIKV surveillance and vector control is essential to combating future epidemics. However, challenges relating to the timely publication of case reports significantly limit the effectiveness of current surveillance methods. In many countries with poor infrastructure, established systems for case reporting often do not exist. Previous studies investigating the H1N1 pandemic, general influenza and the recent Ebola outbreak have demonstrated that time- and geo-tagged Twitter data, which is immediately available, can be utilized to overcome these limitations.

**Methods:**

In this study, we employed a recently developed system called Cloudberry to filter a random sample of Twitter data to investigate the feasibility of using such data for ZIKV epidemic tracking on a national and state (Florida) level. Two auto-regressive models were calibrated using weekly ZIKV case counts and *zika* tweets in order to estimate weekly ZIKV cases 1 week in advance.

**Results:**

While models tended to over-predict at low case counts and under-predict at extreme high counts, a comparison of predicted versus observed weekly ZIKV case counts following model calibration demonstrated overall reasonable predictive accuracy, with an R^2^ of 0.74 for the Florida model and 0.70 for the U.S. model. Time-series analysis of predicted and observed ZIKV cases following internal cross-validation exhibited very similar patterns, demonstrating reasonable model performance. Spatially, the distribution of cumulative ZIKV case counts (local- & travel-related) and *zika* tweets across all 50 U.S. states showed a high correlation (*r* = 0.73) after adjusting for population.

**Conclusions:**

This study demonstrates the value of utilizing Twitter data for the purposes of disease surveillance. This is of high value to epidemiologist and public health officials charged with protecting the public during future outbreaks.

**Electronic supplementary material:**

The online version of this article (10.1186/s12889-019-7103-8) contains supplementary material, which is available to authorized users.

## Background

Zika virus (ZIKV) is an emerging mosquito-borne arbovirus that causes serious public health consequences. Zika virus is primarily transmitted to people through the bite of an infected *Aedes* species mosquito (*Ae. aegypti* and *Ae. albopictus*) [1]. Although most infections carry mild symptoms or are asymptomatic, the association between ZIKV and microcephaly and Guillain-Barré syndrome placed ZIKV as a global medical emergency during the 2016 epidemic [2–6]. Currently there is no medicine or vaccine to cure or prevent ZIKV infection. Therefore, infection containment, vector control and personal protection are the most important measures to prevent infections and contain viral spread [7].

According to the U.S. Centers for Disease Control and Prevention (CDC), ZIKV has been reported in over 60 countries and territories worldwide, during the 2015–2016 ZIKV epidemic, with South America as the most severely affected continent [8]. In the United States, locally acquired ZIKV cases have been reported in Florida and Texas as well as the U.S. territories in Puerto Rico, U.S. Virgin Islands, and American Samoa [8, 9]. Travel-associated U.S. cases of ZIKV infections have been reported in all 50 States in the U.S. [9]. ZIKV can also be sexually transmitted, which suggests concern for potential local outbreaks [9]. By November, 2016, U.S. travel-associated ZIKV cases amounted to 4115. By this time, there were 139 locally acquired mosquito-borne and 35 sexually transmitted cases in the U.S. Cases in U.S. territories amounted to 39,951 [9]. Although there were no reports of microcephaly cases, 13 cases of Guillain-Barré syndrome were reported in the continental U.S., and 50 in U.S. territories [9].

Regarding ZIKV surveillance and vector control, challenges exist that have significantly limited the effectiveness of current methods. In the United States, disease surveillance is supported by the CDC Division of Health Informatics and Surveillance and is carried out through a variety of networks that involve the collaboration of thousands of agencies at the federal, state, territorial, and tribal levels across health departments, laboratories, and hospitals [10–12]. Importantly, while ZIKV cases reported from official sources such as the CDC are of high quality, such reporting is not timely due to an internal protocol of these offices to collect and verify data prior to formal publication. In addition, the cases reported by any single source do not always reflect all the cases that truly exist. More importantly, in many countries or regions with poor infrastructure and healthcare systems, established systems for such case reporting do not exist. To collect as much ZIKV case information as possible with minimal delay, other services are available that can publish such information more timely.

Alternative data sources such as social media and other digital services provide an opportunity to overcome existing surveillance obstacles by providing relevant information that is temporally and geographically tagged. To date, the variety of digital data streams that have been utilized to help track diseases over time and space have included internet search engines [13–18], electronic health records [19], news reports [20, 21], Twitter posts [22–26], satellite imagery [27], clinicians’ search engines [28], and crowd-sourced participatory disease surveillance systems [29–31].

In terms of social media data streams, Twitter is a free social networking service that enables millions of users to send and read one another’s brief messages, or “tweets,” each day. Tweets can be posted either publicly or internally within groups of “followers.” Currently, this service includes approximately 326 million registered users, with 67 million in the United States [32]. In spite of a fair amount of noise due to general chatter and the sheer number of tweets, Twitter contains useful information that can be utilized for disease surveillance and forecasting.

Previously, Twitter has been utilized to measure public anxiety related to stock market prices, national sentiment, and the impacts of earthquakes [33–35]. More recently, Twitter was used in epidemic tracking and forecasting for the H1N1 pandemic, general influenza, and the recent Ebola outbreak [25, 36–39]. In terms of ZIKV, studies have made use of Twitter data and developed predictive models for a variety of applications. Mandal et al. (2018) developed Twitter-based models to track zika prevention techniques and help inform health care officials [40]. Other studies have performed content analysis of Twitter data to explore and predict what types of zika-related discussions people were having during the recent ZIKV epidemic [41–44].

Since ZIKV outbreaks are influenced by many environmental and social factors, such as local mosquito species and density distributions, season, climate, land use, land cover, human demographics, and mitigation efforts, successful surveillance and forecasting of the disease can be difficult [45–51]. Use of live streaming ZIKV-related information via nationwide tweets could represent a practical, timely, and effective surveillance tool, in turn improving ZIKV case detection and outbreak forecasting [14, 52]. To date, however, studies making use of Twitter data to monitor the spread of ZIKV in real time and space have been limited.

In one study, Teng et al. (2017) developed models to forecast cumulative ZIKV cases [13]. However, these models were developed to predict ZIKV cases cumulatively, and on a global basis. Further, these studies did not make use of the Twitter data stream, but rather Google Trends. Similarly, Majumder et al. (2016) developed models to forecast ZIKV case counts in Columbia during the recent ZIKV epidemic [14]. Again, however, the analysis utilized Google Trends data and considered cumulative, rather than weekly, case counts. In our research, we identified only a single study that attempted to forecast ZIKV using the Twitter data steam, and on a weekly basis. In this study by McGough et al. (2017), the authors demonstrated the utility of developing Twitter-based models to forecast ZIKV in countries of South America [26]. However, given the lack of robust diagnostic capabilities in the region, the study was limited to using “suspected,” rather than “confirmed,” ZIKV cases. Additionally, the study did not examine spatial patterns of ZIKV cases and tweets, nor did it compare local- versus national-level modeling. To the best of our knowledge, there has been only a single study to date to harness digital data streams for near-real time weekly forecasting of ZIKV cases, and no such study to date that has utilized Twitter data for ZIKV forecasting in the United States and offered a comparison of national- and state-level models [26].

In this study, we demonstrate the value of utilizing time- and geo-tagged information embedded in the Twitter data stream to 1) examine the relationship between weekly ZIKV cases and ZIKV-related tweets temporally and spatially, 2) assess whether Twitter data can be used to predict weekly ZIKV cases and, if so, 3) develop weekly ZIKV predicative models that can be used for early warning purposes on a state and national level. This study contributes to the body of literature.

## Methods

### Twitter data

We utilized a general-purpose system called Cloudberry to filter a 1% random sample of U.S. Twitter data. Cloudberry is a subscription client of the Twitter stream application program interface (API). It is connected to the big data management system Apache AsterixDB, which allows for the efficient transformation of front-end data requests, and the continuous ingest and storage of data [53]. In the Cloudberry system, the geographic location filtering parameters are set by a rectangular bounding box that includes all U.S. territory. Since this boundary box also covers Canada and parts of Mexico, tweets that are not published in the U.S. are deleted. Cloudberry enables interactive analytics and visualizations of large amounts of data containing temporal, spatial, and textual components common to Twitter and other social media applications. Data collection from Cloudberry began on November 17, 2015, with approximately one million tweets stored per day, and over 874 million tweets collected so far. The Cloudberry system enables the filtration of millions of tweets according to specific parameters set by the API user.

Live tweet counts from Cloudberry were collected using Twitter’s open streaming API, which has been shown to be representative of Twitter’s greater information database and therefore useful for research purposes [54–56]. The Streaming API can take three parameters; namely, keywords (words, phrases, or hashtags), geographical boundary boxes, and user ID. For the first two parameters, in order to identify and compile tweets that were relevant to ZIKV, we filtered data for the entire U.S. and for Florida during the year 2016 using the keywords *zika* and *mosquito*. The latter keyword was employed to explore whether it could provide an early warning signal of impending ZIKV activity. For the third parameter, no user ID was specified so as not to restrict sample size. Tweet counts were summed by week in order to be used in weekly prediction models. In using Twitter data for this study, we complied with Twitter’s terms, conditions, and privacy policies.

### ZIKV data

Updated case reports for total U.S. ZIKV were available on a weekly basis throughout the duration of the 2016 ZIKV epidemic. Data was obtained using the Morbidity and Mortality Weekly Reports maintained by the CDC [57]. For cumulative state-by-state ZIKV prevalence data, we accessed an updated CDC online report on January 24, 2017 [9]. ZIKV cases for the state of Florida were available approximately every day during the ZIKV epidemic, and were obtained via the Florida Department of Health [58].

The Florida Department of Health reports only cumulative case counts. To convert this to weekly case counts for use in this study, we simply took the difference of cumulative cases from 1 week to the next. All ZIKV case counts used in this study were based on date of reporting.

### Statistical analysis

#### Temporal correlation

Time-series analysis was conducted for weekly ZIKV cases and *zika* tweets to illustrate their patterns over the 2016 study period. This was also conducted for *mosquito* tweets. To assess the correlation of weekly *zika* tweets with weekly ZIKV cases, we produced Pearson correlation coefficients. To assess the potential lag in time between ZIKV cases and each tweet keyword, we examined the change in these coefficients after applying lags ranging from 0 to 6 weeks. This time range takes into account the approximately 1–2 week incubation period of ZIKV as well as the potential 2–4 week delay between ZIKV laboratory testing and reporting [59, 60].

#### Spatial correlation

The cumulative prevalence of ZIKV cases was also examined and correlated (using Pearson correlation) with cumulative *zika* tweets spatially across the U.S. Results were depicted in the form of two maps, each divided into four shaded quartiles. For this analysis, cases and tweets for each state were calculated as the sum of cases and tweets from January 1, 2016 through January 24, 2017. Cases were depicted as raw case counts. However, for proper spatial comparison, cumulative tweets were adjusted according to state population, using 2016 U.S. Census Bureau population estimates [61]. Therefore, tweets per 100,000 people were reported, and referred to as tweet prevalence. Cumulative data was calculated through January 24, 2017 because the CDC only provides cumulative state-by-state ZIKV prevalence data for the date that data is accessed, not historically. That is, the CDC maintains an online report that is updated each week, at which point historical numbers are no longer available [9]. In our case, we accessed the CDC website on January 24, 2017. This additional 24 day period is unlikely to have impacted our analysis as the ZIKV epidemic had dramatically slowed by this point, adding relatively few additional cases.

#### Model development

Univariate analyses using 1) weekly ZIKV case counts lagged by 1 week and 2) weekly *zika* tweet counts lagged by 1 week as predictors of weekly ZIKV case counts in Florida and the U.S. was first carried out. This was to assess the potential utility of using Twitter data where quantitative ZIKV case reporting is not reliable, as well as to understand the extent to which each term alone is predictive of future case counts.

Next, prediction models combining both prior ZIKV case counts and Twitter data to estimate weekly ZIKV case counts were explored. Specifically, we applied an auto-regressive (AR) model using *zika* tweet counts as an input series. Two types of covariates were used; namely, prior weekly ZIKV case counts and prior weekly *zika* tweet counts. Prior to model development, first-order differencing was applied to both dependent and independent variables. This is standard practice to address the issue of stationarity that is common to time-series data. After differencing, we examined various models using 1–6 week lags [AR(1,1)-AR [1, 6]] for both the auto-regressive variable as well as the tweet variable according to the following general equation:

1 $$ {\mathrm{ZIKV}}_{\mathrm{t}}^{\prime }=\upalpha +\sum \limits_{\mathrm{k}=1}^6{\upbeta}_{\mathrm{k}}{\mathrm{ZIKV}}_{\mathrm{t}-\mathrm{k}}^{\prime }+\sum \limits_{\mathrm{k}=1}^6{\upgamma}_{\mathrm{k}}{\mathrm{Tweet}}_{\mathrm{t}-\mathrm{k}}^{\prime }+\upvarepsilon $$

where ${\mathrm{ZIKV}}_{\mathrm{t}}^{\prime }$ is the difference between the ZIKV case count on week t and week t-1 (first-order difference); β_k_ is the effect estimate of the weekly ZIKV case count k week(s) prior to t after first-order differencing; γ_k_ is the effect estimate of the weekly *zika* tweet count k week(s) prior to t after first-order differencing; α is the regression intercept; and Ɛ is the error term.

In order to select an appropriate predictive model for Florida and a model for the U.S., several steps were taken. First, candidate models were identified. A model was considered a candidate model only if all predictor terms were significant at *p* < 0.05 and if auto-correlation of residuals passed the white noise test (*p* > 0.05). Candidate models were then compared and the model with the lowest Akaike Information Criterion (AIC) value was selected as the final model (one model for Florida and one for the U.S.). Using the AIC criterion ensured that the chosen models were not over fit. Final models were also evaluated to ensure normality of residuals.

In testing models, two highly inflated weekly *zika* tweet counts that occurred well before the major onset of the ZIKV epidemic, and which could be explained by high profile media events, were reduced to the mean of their before and after values. These inflated points occurred during the first 2 weeks of February, coinciding with the timing of the first ZIKV cases reported in the United States by the CDC (week of Jan. 30). Additionally, the World Health Organization (WHO) officially declared a ZIKV public health emergency of international concern that same week (Feb. 1) followed by an announced request by President Obama the following week (Feb. 8) for $1.8 billion in ZIKV-related emergency funds [62]. Inflation of these points was accounted for prior to analysis to prevent this prominent media activity from influencing model coefficients. Final models were also regressed with the inclusion of these original values to ensure minimal model sensitivity.

After model calibration, both predicted and measured weekly ZIKV case counts for each model were plotted for comparison.

### Model evaluation

To validate the models we applied forecast evaluation with a rolling origin, which is a form of leave-one-out cross-validation. That is, a model was fit to all but one (left out) weekly data point. The fitted data was then used to predict the left out data point. This process was repeated 52 times (for all weeks), with each iteration holding out a new weekly data point. This process generated a new data set composed entirely of predicted weekly ZIKV case counts for each model. The predicted and measured values for these aggregated datasets were then plotted in the form of two scatter plots (one for the Florida model and one for the U.S. model) as well as two time-series plots. Goodness of fit for predictions was assessed using the coefficient of determination (R^2^) and root mean squared error (RMSE) for the scatter plots of predicted and measured weekly ZIKV case counts.

## Results

### Temporal correlation

**Fig. 1:**
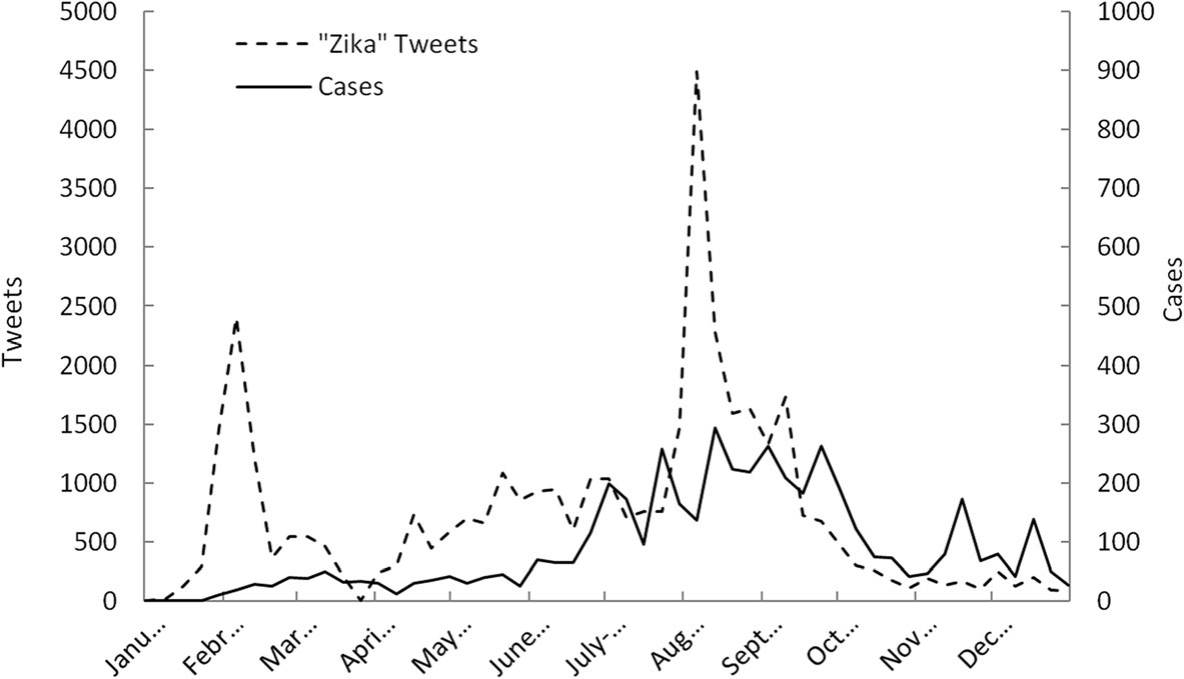
Total ZIKV cases and *zika* tweets during the year 2016 in the United States

Figure 1 is a time-series plot of total (local- and travel-related) ZIKV cases and *zika* tweets occurring in the United States for each week during the year 2016. As shown, the pattern of case reports and tweets was very similar, exhibiting a gradual increase in both tweets and cases during the spring months, with a prominent peak occurring during summer months. An increase of ZIKV cases during summer months is consistent with the primary mode of ZIKV transmission, namely mosquito bites, since mosquitoes are more prevalent in warmer, humid conditions. After the summer months, tweets and cases both declined. A prominent spike in *zika* tweets that did not coincide with ZIKV cases was apparent in the first half of February. This peak coincides with the occurrence of the high profile media events previously described; namely, reports of the first ZIKV cases in the U.S., as well as the public health emergency announcement by the WHO and the ZIKV-related emergency funds requested by President Obama. In examining the relationship between weekly ZIKV cases and weekly *zika* tweets during the study period, applying a 1 week lag term for *zika* tweets resulted in a better correlation (r = 0.67) when compared with either no lag (r = 0.51) or greater lag periods (r = 0.50–65).

**Fig. 2:**
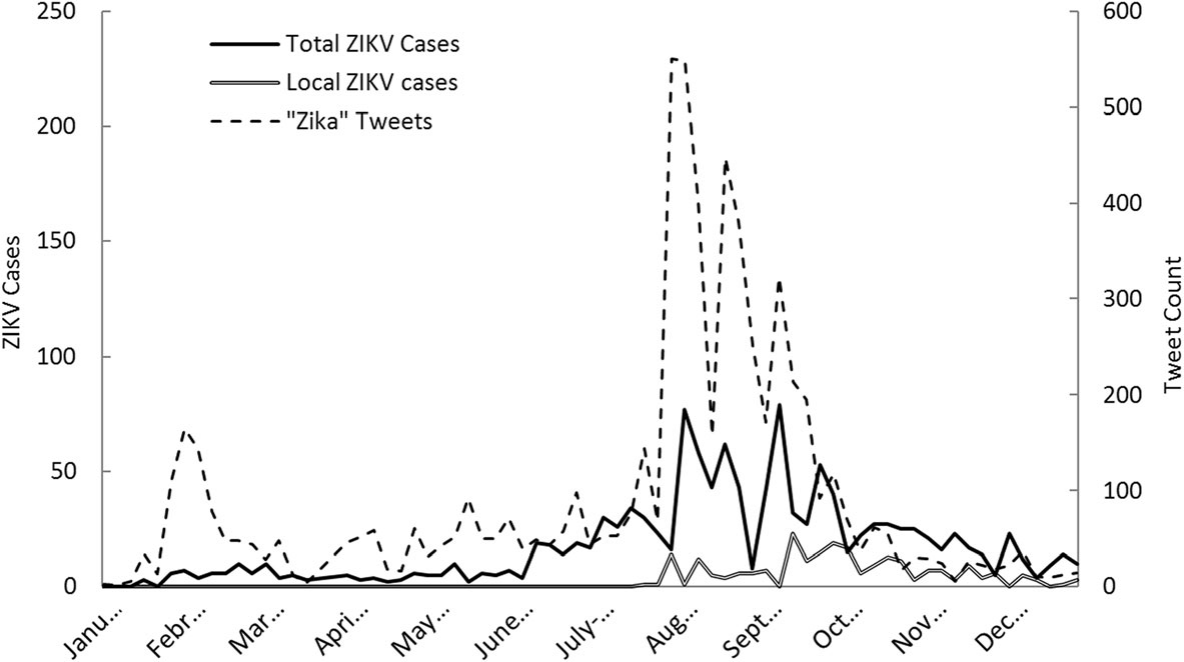
Weekly ZIKV cases and *zika* tweets during the year 2016 in Florida

Figure 2 is a time-series plot of total (local- and travel-related) ZIKV cases and *zika* tweets occurring in Florida for each week during the year 2016. As with Fig. 1, the pattern of case reports and tweets was very similar, exhibiting a strong increase during the months of July, August, and September. Tweet counts exhibited a trimodal distribution during the peak of the outbreak. A very similar pattern was apparent for ZIKV cases. Locally acquired ZIKV was not reported until the end of July and peaked during the month of September, after which the pattern of decline followed a similar trajectory as total ZIKV cases.

In examining Fig. 2, we observed a sharp increase in *zika* tweets that predated the increase in total ZIKV cases by 1 week. In examining the relationship between weekly ZIKV cases and weekly *zika* tweets during the study period, applying a 1 week lag term for *zika* tweets improved the correlation from 0.64 (zero lag) to 0.77. As with Fig. 1, a peak in tweets during February coincided with the previously described high media activity.

Use of *mosquito* tweets was explored for its potential to serve as an advanced warning signal for impending rises in ZIKV cases. A very similar pattern in tweet frequency existed between the *mosquito* and *zika* keywords. A time-series plot of weekly *zika* tweets and *mosquito* tweets occurring in Florida in 2016 is presented in Additional file 1: Figure S1. The correlation between weekly *zika* and *mosquito* tweets during 2016 was 0.87 (*p* < 0.001), which is considerably high. Use of the keyword *mosquito* therefore provided no added benefit as a temporal indicator of ZIKV compared to the keyword *zika*. This was most apparent during the peak of the outbreak, when both keywords responded nearly identically over time.

### Spatial correlation

**Fig. 3:**
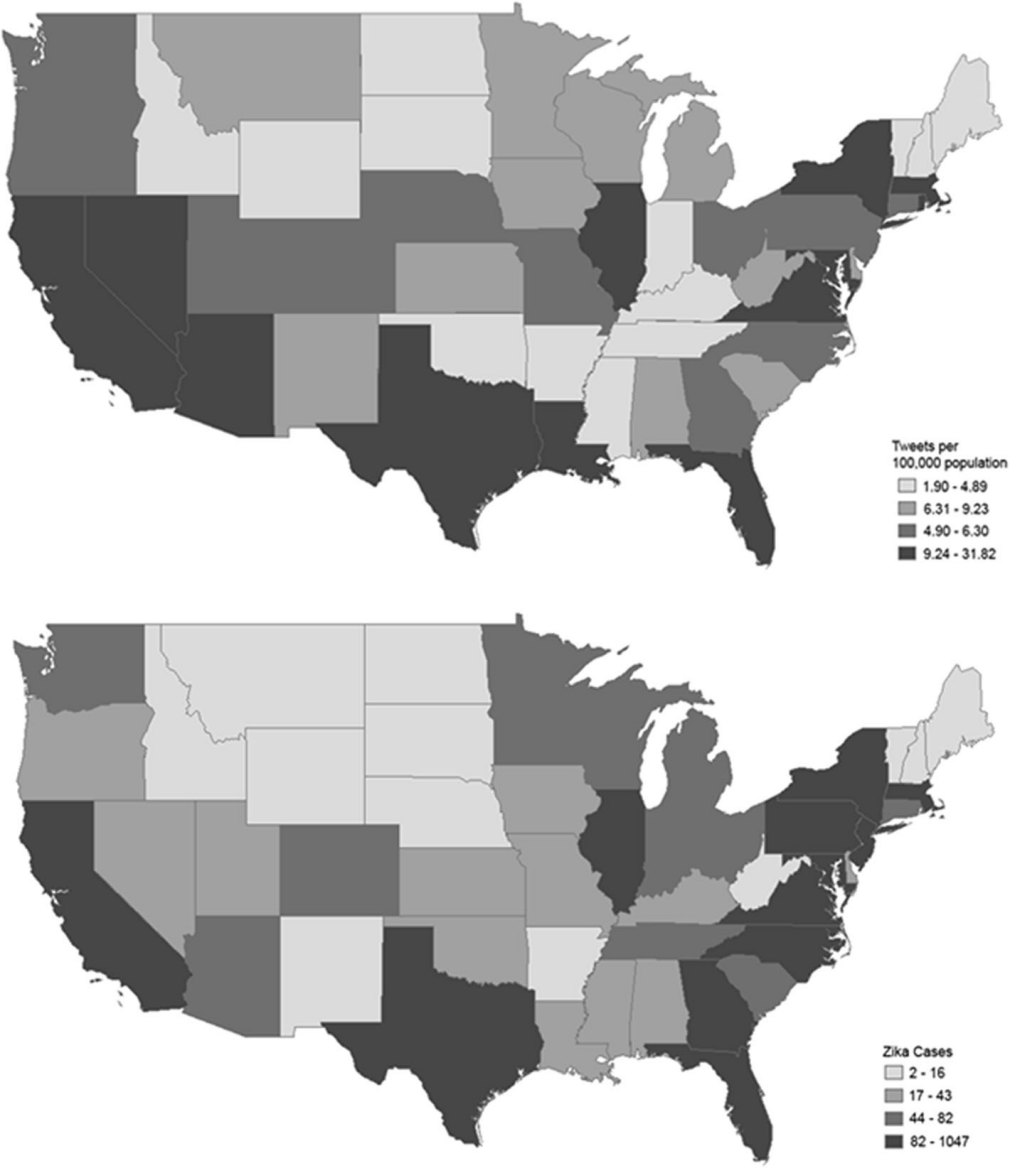
Comparison of cumulative ZIKV cases and population-adjusted *zika* tweets for approximately 1 year (2016) in the United States

Figure 3 depicts the cumulative prevalence of ZIKV cases and *zika* tweets by state, from January 1, 2016 through January 24, 2017. Cases are presented as case counts, whereas tweets are presented as tweets per 100,000 people. As shown, states with the highest prevalence of tweets and cases (darkest shade) showed high similarity. A Pearson correlation coefficient of 0.73 (*p* < 0.001) was produced when assessing cases and population-adjusted tweets across all 50 states.

Of the ten states with the most ZIKV cases, seven also had the highest prevalence of *zika* tweets. In order of descending case count, these states included Florida, New York, California, Texas, Maryland, Massachusetts, Virginia, and Illinois. States that were in the top quartile for tweets, but not for ZIKV cases, were states that were geographically adjacent (shared border) to states with the highest ZIKV case counts. Such states included Louisiana, Nevada, and Arizona. Of the ten states with the fewest ZIKV cases, six also had the lowest *zika* tweet prevalence. Regions with fewest ZIKV cases and tweets were the upper Midwest (Idaho North Dakota, South Dakota, and Wyoming) and Northeast (New Hampshire, Vermont, and Maine).

### Prediction model

The model chosen to predict ZIKV case counts in Florida was an AR [1, 3] model using a one-week lag for *zika* tweets. That is, the model included a term for weekly ZIKV case counts one, two, and 3 weeks prior (${\mathrm{ZIKV}}_{\mathrm{t}-1}^{\prime }$, ${\mathrm{ZIKV}}_{\mathrm{t}-2}^{\prime }$, and ${\mathrm{ZIKV}}_{\mathrm{t}-3}^{\prime }$) as well as a term for weekly *zika* tweets 1 week prior (${\mathrm{Tweet}}_{\mathrm{t}-1}^{\prime }$). Models tested without a term for prior *zika* tweets exhibited a lower AIC when compared to candidate models that used tweet information. This suggests that Twitter data improved the predictive ability of the model. Additionally, models that used fewer AR terms resulted in a higher AIC, suggesting that the use of multiple AR terms did not produce a model that was overfit.

For U.S. predictions, a similar model was chosen, but with one fewer auto-regressive term [AR [1, 2]]. That is, the model included a term for weekly ZIKV case counts one and 2 weeks prior (${\mathrm{ZIKV}}_{\mathrm{t}-1}^{\prime }$ and ${\mathrm{ZIKV}}_{\mathrm{t}-2}^{\prime }$) as well as a term for weekly *zika* tweets 1 week prior (${\mathrm{Tweet}}_{\mathrm{t}-1}^{\prime }$). As with Florida, models tested without a term for prior *zika* tweets had a lower AIC than those that included tweet counts, again suggesting an improvement of the model when using Twitter data. And similarly, models that used alternate number of AR terms had higher AICs, suggesting a less appropriate model.

Table 1 presents regular effect estimates and standardized estimates for covariates, along with standard errors, and *p*-values for the multivariate and univariate models calibrated in Florida and the U.S. The R^2^ value for the fit of observed versus predicted weekly ZIKV case counts following calibration for each model is also presented. All covariates in the presented models were significant at *p* < 0.05. Intercept effects were not significant, thus contributing little to the models. This is expected since first-order differencing was applied (mean should approximate zero).

**Table 1:** Output for ZIKV predictive models

		Effect Estimate	Standardized Effect Estimate	Standard Error	*P*-Value	Model R^2^
Florida Models						
Multivariate	Intercept	0.2750	0.0013	0.6350	0.6670	0.74
	ZIKV_t-1_	− 0.6993	−0.6993	0.1352	< 0.0001	–
	ZIKV_t-2_	−0.6271	−0.6271	0.1432	< 0.0001	–
	ZIKV_t-3_	−0.4264	−0.4264	0.1373	0.0033	–
	Tweet_t-1_	0.0626	0.4104	0.0136	< 0.0001	–
Univariate	Intercept	0.2711	0.0007	2.1455	0.9000	0.60
	Tweet_t-1_	0.0443	0.2903	0.0211	0.0408	–
Univariate	Intercept	0.2720	−0.0002	1.5694	0.8631	0.61
	ZIKV_t-1_	−0.3282	−0.3281	0.1350	0.0187	–
U.S. Models						
Multivariate	Intercept	1.0587	0.0107	3.4241	0.7586	0.70
	ZIKV_t-1_	−0.5221	−0.5221	0.1402	0.0005	–
	ZIKV_t-2_	−0.3806	−0.3806	0.1457	0.0120	–
	Tweet_t-1_	0.0242	0.2622	0.0114	0.0392	–
Univariate	Intercept	0.4715	0.0001	7.2653	0.9485	0.63
	Tweet_t-1_	0.0325	0.3517	0.0123	0.0114	–
Univariate	Intercept	0.2720	0.0023	1.5694	0.8631	0.64
	ZIKV_t-1_	−0.3282	−0.3756	0.1350	0.0187	–

*Note, effect estimates represent the effects of covariates after first-order differencing; thus explaining the negative coefficients of AR terms that are otherwise positively auto-correlated

AIC values for the two chosen multivariate models as well as all candidate models and the univariate models are presented in Additional file 1: Table S1. Additional diagnostic criteria including the white noise test and partial auto-correlation functions for each model are presented in Additional file 1: Figures S2 and S3. Residuals plots are presented in Additional file 1: Figures S4 and S5, and showed that the models tended to over-predict at low case counts and under-predict at high counts. Lastly, Additional file 1: Table S2 presents zero mean test results, allowing us to affirm that no unit root exists and that the data series used in this analysis is stationary.

**Fig. 4:**
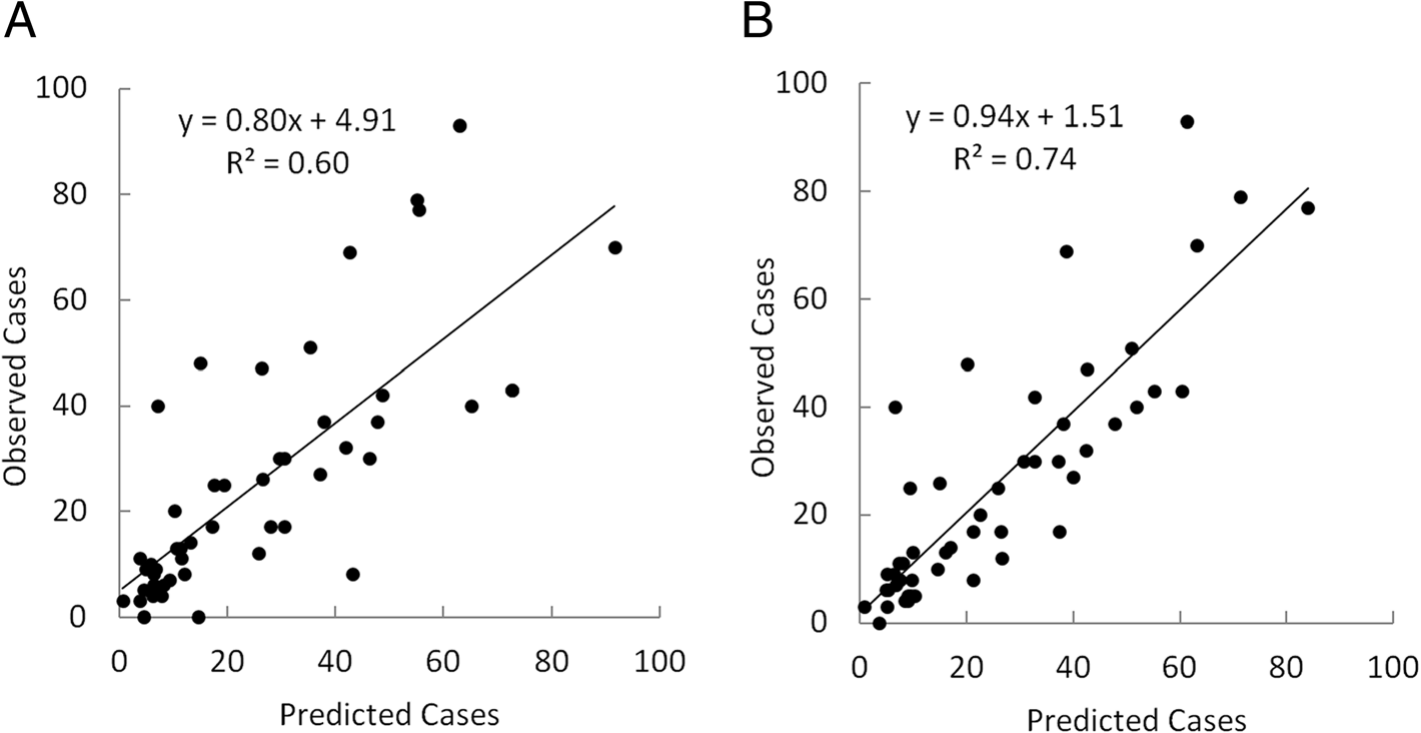
Relationship between predicted and measured weekly ZIKV case counts during 2016 in Florida after calibrating a model using **a**) only Twitter data and **b**) Twitter data plus prior ZIKV case reports

Figure 4 depicts predicted versus measured weekly ZIKV case counts after calibrating the univariate model using only Twitter data (Fig. 4a) and the multivariate model (Eq.1) using Twitter data and prior ZIKV case counts (Fig. 4b) for the state of Florida. Models were calibrated using 52 weekly data points. However, since forecasts required 3 weeks of prior data, only 49 points could be predicted and plotted. Results for predicted and observed case counts in Florida using the univariate model (Fig. 4a) demonstrated that Twitter data alone can be a useful predictor of weekly case counts (*R*^2^ = 0.60), predicting about as well as prior ZIKV case counts (R^2^ = 0.61). However, Fig. 4b demonstrated that combining prior ZIKV case counts and Twitter data results in a substantially improved model, with a higher R^2^ of 0.74 (RMSE = 11.7 cases). The combined model using prior ZIKV and Twitter data suggests good predictive ability. Plotting predicted and observed case counts following cross-validation of the multivariate model produced an R^2^ of 0.67 and RMSE of 13.3 cases. This further indicates reasonable performance of the model considering that in this case no information from the plotted points was used in model calibration.

**Fig. 5:**
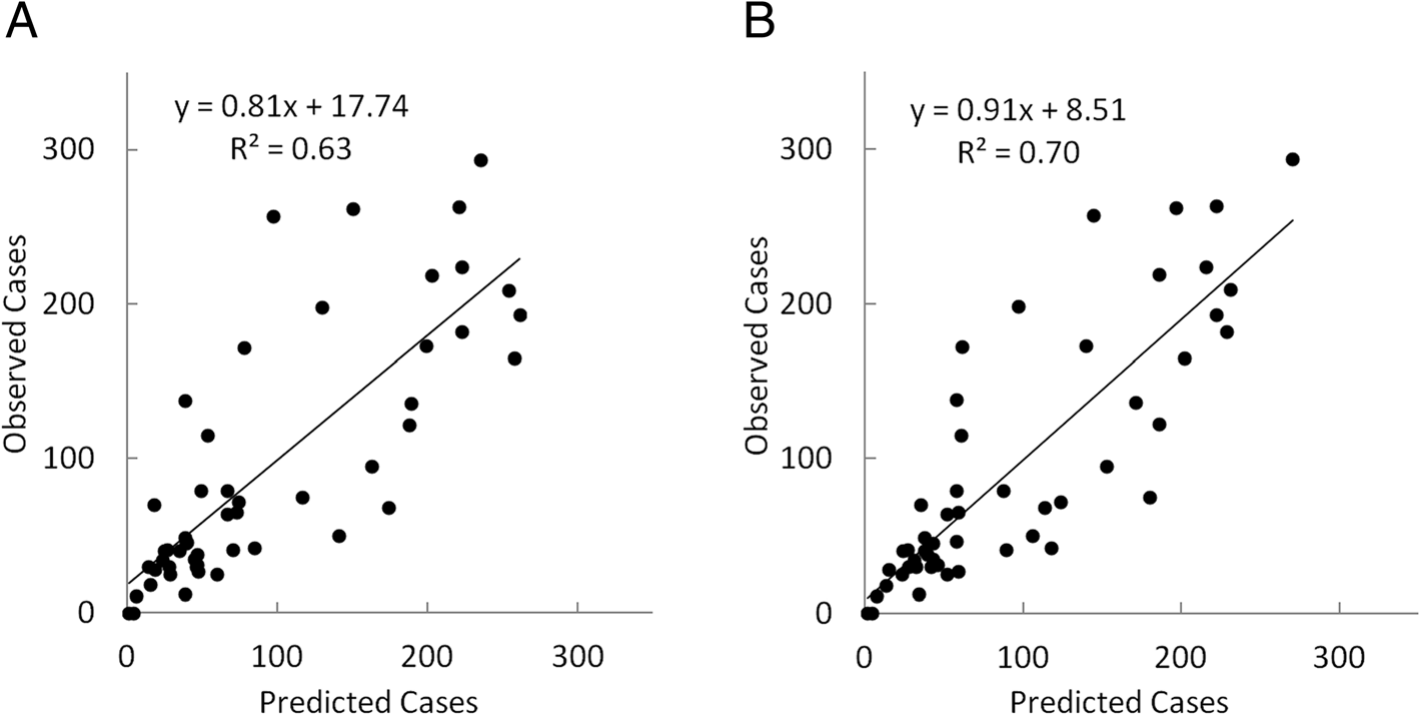
Relationship between predicted and measured weekly ZIKV case counts during 2016 in the United States after calibrating a model using **a**) only Twitter data and **b**) Twitter data plus prior ZIKV case reports

Figure 5 is similar to Fig. 4, depicting predicted versus measured weekly ZIKV case counts according to a univariate model (Fig. 5a) and multivariate model (Fig. 5b). In this case, however, the U.S. model was applied using data for the entire nation. Similarly, results for predicted and observed case counts using the univariate model (Fig. 5a) demonstrated that Twitter data alone can be a useful predictor of weekly case counts (R^2^ = 0.63); again predicting about as well as the univariate model using prior ZIKV case reports. However, in Fig. 5b we again observed that combining prior ZIKV case counts and Twitter data led to model improvement, with a higher R^2^ of 0.70 (RMSE = 44.5 cases). Following internal cross-validation of the multivariate model, predicted and observed case counts in the U.S. resulted in an R^2^ of 0.57 and RMSE of 54.2 cases. This suggests that the Florida model performed better following validation than the U.S. model. Upon elimination of a single outlier prediction in October, however, validation results for the U.S. model improved markedly, with an R^2^ of 0.63 and RMSE of 49.4 cases.

**Fig. 6:**
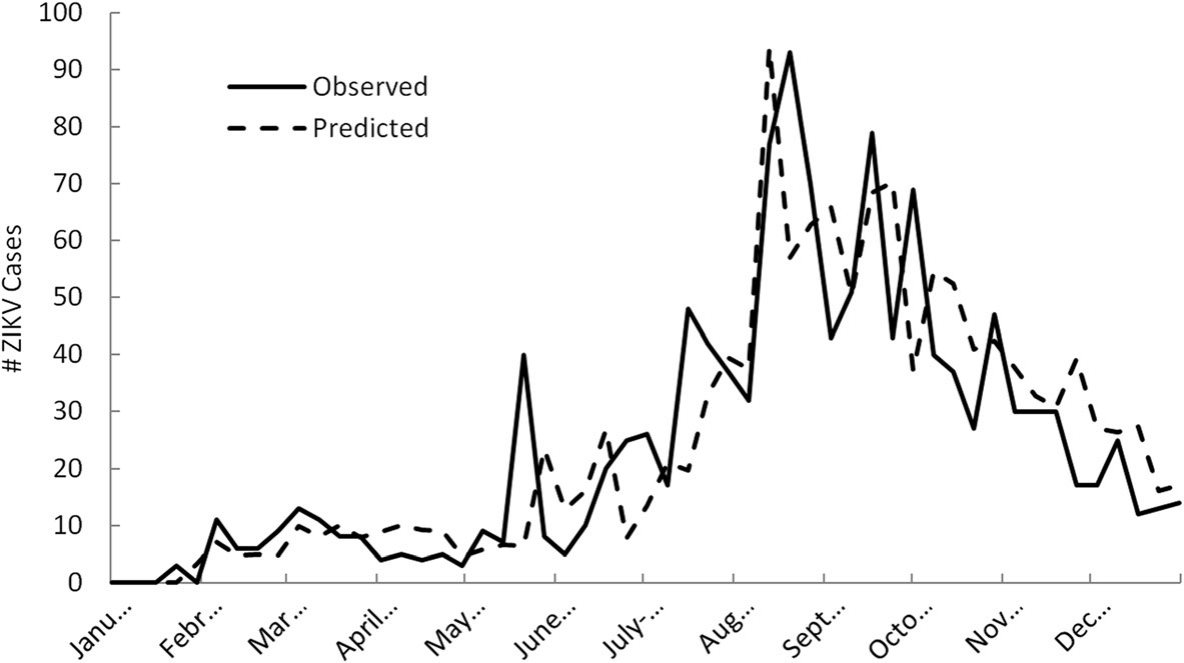
Time-series plot using cross-validation results of observed and predicted weekly ZIKV case counts during 2016 in Florida

Figure 6 shows a time-series plot using cross-validation results of observed and predicted weekly ZIKV case counts during 2016 using the multivariate Florida model. Since validation results were used, none of the predicted data shown in this plot was incorporated in the model calibration process. Rather, each weekly prediction represents the single held out point that was predicted on during each iteration of the validation process. As shown, a very similar pattern in weekly predicted and observed case counts exists over time. The general increase in case counts during spring, followed by a summertime peak, and subsequent decline in the fall is predicted well by the model. The two major outbreak peaks observed during summer were also predicted well in terms of magnitude and time of onset. However, in the case of the largest peak, the duration of this major outbreak period was under-predicted slightly. The correlation between predicted and measured weekly ZIKV case counts was high, with a correlation coefficient of 0.82 (*p* < 0.001) over the study period.

**Fig. 7:**
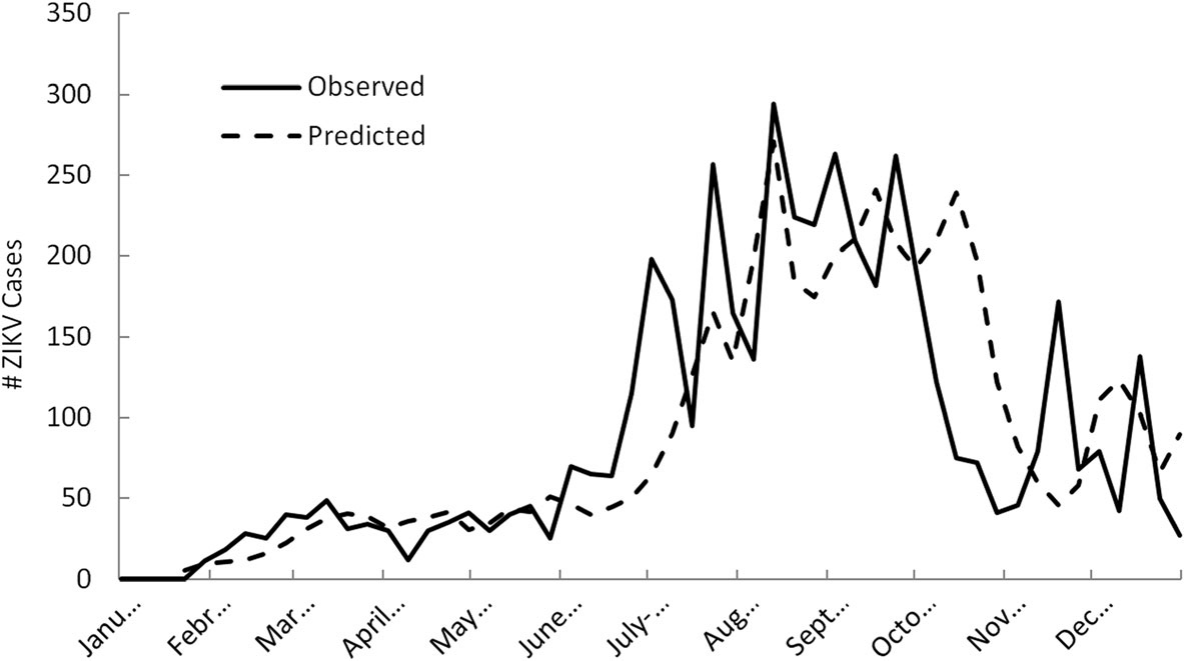
Time-series plot using cross-validation results of observed and predicted weekly ZIKV case counts during 2016 in the United States

Figure 7 shows a time-series plot of observed and predicted weekly ZIKV case counts during 2016 in the U.S using the nationally calibrated multivariate model. Similar to Fig. 6, predictions represent results from the internal cross-validation process. As shown, the national model predicted the general increase in case counts during spring, followed by a summertime peak, and subsequent decline in the fall. However, the model missed the onset of the first major peak in summer case counts and under-predicted the second peak. The third and highest peak that occurred in August, however, was predicted well by the model in terms of timing, magnitude, and duration. A subsequent summertime peak was predicted prematurely, followed by a false prediction peak. Overall, the correlation between predicted and measured weekly ZIKV case counts was still high, with a correlation coefficient of 0.75 (*p* < 0.001) for the 2016 study period.

## Discussion

Weekly ZIKV case reports and *zika* tweets in the U.S. and in Florida exhibited very similar temporal patterns, peaking during summer and declining in fall. A multivariate auto-regression analysis using Florida and U.S. data demonstrated *zika* tweets to be an important predictor of weekly ZIKV case counts during the 2016 study period. Combined with information of previous ZIKV case counts, we calibrated two models that were able to estimate weekly ZIKV cases 1 week in advance with reasonable accuracy; one model for Florida and one model for the U.S. Both models performed best when both prior ZIKV case count data and Twitter data were included. Following calibration of the models, and subsequent internal cross-validation, a comparison of predicted versus observed weekly ZIKV case counts demonstrated reasonable model performance for the Florida model and reduced, but still moderate, performance for the national model. A time-series plot of predicted and observed case counts similarly showed the Florida model to predict reasonably well and the national model to predict moderately well. While a comparison of observed and model-predicted ZIKV case counts produces R^2^ values ≥0.70 for the Florida model, we must be careful not to overstate the model performance given that disease forecasting models can sometimes yield R^2^ > 0.9. Nonetheless, results for both models in this study suggest that Twitter data can be used to help track ZIKV prevalence during outbreak periods. Given that Twitter data is immediately available, compared to a delay of cases often reported by the CDC, Twitter represents a particularly useful tool for epidemiologist and public health officials involved in disease surveillance.

During model development, 1 week lagged *zika* tweets were best correlated with weekly ZIKV cases. This is visually apparent during the major outbreak period in Florida, where a sharp rise in *zika* tweets appeared to precede ZIKV cases by 1 week. A possible explanation is that an inherent temporal difference exists between Twitter chatter and ZIKV diagnosis. For instance, it is plausible that discussion of ZIKV (potentially due to the presence of symptomatic or hospitalized family members or friends) predates actual diagnosis. In this case, a rise in *zika* tweets would predict a rise in ZIKV cases. Whether or not this temporal difference in *zika* tweets is truly reflecting chatter related to the impending rise in ZIKV cases, however, cannot be confirmed here.

It is worth noting that reports of the first locally acquired ZIKV in Florida corresponded with the sharp rise in *zika* tweets occurring in August. Therefore, an alternative explanation is that the initial sharp rise in *zika* tweets occurring in summer could reflect chatter related to the first few cases of locally acquired ZIKV, rather than the impending increase in total ZIKV cases that occurred the following week. This explanation, however, fails to explain the overall higher correlation between ZIKV case counts and 1 week lagged *zika* tweets over the entire study period.

The primary strength of these models is the use of readily available, real-time Twitter data to estimate ZIKV cases. Additionally, the use of 1 week old ZIKV case reports to generate a good estimate means reduced dependence on the timely publication of case reports by government agencies in order to track ZIKV and predict outbreak trends. Where states report case counts on a daily basis during an outbreak (e.G. *Florida*), estimates of the following week’s ZIKV case counts can similarly be updated on a real-time (daily) basis. This enables better epidemic preparedness by local and state public health agencies in charge of disease response.

A primary limitation of these models is the need for historical ZIKV case count information. This requires the government to continue monitoring and publishing case reports. Though such surveillance takes place in the U.S. and other industrialized nations, it does not take place in many developing countries. Furthermore, government data may not always be released in time to enable ZIKV case predictions. In such regions where quantitative case count data is not accurately and/or consistently reported, or potentially delayed, univariate analyses using only prior *zika* tweets demonstrated that Twitter data may still be useful for disease surveillance. This assumes that Twitter is used among the local population and that sufficient knowledge of the disease and disease activity exists among the population.

Also noteworthy, since these are statistical models that depend on previous case reports, they cannot be used to predict a ZIKV outbreak where no prior case reports exist. Additionally, given their dependence on historical trends, these models are limited in their ability to predict historically anomalous events that could give rise to dramatic changes in disease prevalence. To this end, mechanistic models that take into account meteorology, vector distribution, population distribution and movement would provide more insight. Also, a diagnosis issue related to the cross-reactivity of diagnostic assays with other arboviruses presents a unique challenge for ZIKV surveillance. This challenge exists with traditional surveillance methods and is still an issue using our modeling approach.

Residuals plots for our models exhibited a departure from normality, with models tending to over-predict at low case count values and under-predict at high case counts. The models’ capability in predicting the full range of cases is compromised because of its over-prediction at extremely low values and under-prediction at extremely high values. Although this tendency toward extreme value prediction is quite common in statistical predictive models trained based on a limited number of measurement data, it nonetheless represents a limitation in this type of statistical modeling that needs to be acknowledged.

Importantly, this work presents predictive models designed with the goal of using covariates to forecast an outcome variable; namely, ZIKV cases. This is distinct from explanatory modeling, which seeks to understand the causal relationships between covariates and outcome variables [63]. In this study, we do not pursue such causal inference. Therefore, while *zika* tweets serve useful in predicting ZIKV cases, we do not make claims about the relationship between tweets and ZIKV cases.

Understanding why *zika* tweets correlate well with ZIKV case counts and therefore offer utility as a surveillance tool is an interesting question. It is possible that *zika* tweets are capturing tweets related to first-hand illness, or that such tweets are merely capturing ZIKV awareness, or a combination of both. While this is an area of active research, the lack of a complete understanding of this relationship does not prevent *zika* tweets from serving as a useful predictor variable in the development of ZIKV forecasting models.

In discussing this study, it is important to avoid ‘big data hubris’ [64]. That is, while our models demonstrate the ability of Twitter data to serve as an indicator of disease activity, such data should not be viewed as a substitute for traditional data collection and analysis, but rather a supplement to such traditional approaches. In future work, combining Twitter data with traditionally collected data related to vector population density, vaccine injection, transmissibility, and basic reproductive number would be useful to incorporate into modeling efforts.

A prominent, temporary spike in tweets that did not coincide with ZIKV cases occurred in early February. This was visible in total U.S. data and Florida data. Such a spike was months ahead of actual major ZIKV activity in the U.S. and can be explained by several important media-related occurrences. This time period marked the occurrence of the first cases of ZIKV to be reported in the United States by the CDC (week of Jan. 30). Of additional relevance was the WHO having declared a ZIKV public health emergency of international concern (Feb. 1) and President Obama announcing a request for $1.8 billion in ZIKV-related emergency funds the following week (Feb. 8). This was a very high profile week for ZIKV in terms of media attention. The inflation of such tweets by these respective events was reflected in actual Twitter content. A qualitative content analysis of trending ZIKV-related topics during this time period supported the existence of particular concern among the population over the arrival of ZIKV to the U.S., showing an overwhelming prevalence of such tweets as “Zika Health Emergency,” “Zika Virus is in the US!,” and “Great, Zika cases here.” Additionally, tweets that included “#CDC” were 2–4 times higher during the period when these events took place than during any week over the following 3 month period. Since these instances of media-related tweet inflation were infrequent, they did not appear to impact our predictive models. In using Twitter data for disease surveillance in the future it is nonetheless important for researchers to be mindful of the influence such major media headlines can have on tweet count, so as not to infer disease.

Two other points of deviation between tweet counts and ZIKV cases occurred during the months of November and December. In these cases, ZIKV cases increased sharply without corresponding increases in *zika* tweets. A possible explanation for this is the announced ending of the ZIKV public health emergency on November 18th by the WHO [65]. This announcement potentially relieved public concern of ZIKV, which may have in turn depressed *zika* tweets in the weeks following.

When comparing national versus Florida ZIKV cases and tweets, time-series analyses showed national tweets to increase more dramatically during the major outbreak period, responding less to weekly vicissitudes in case counts (Figs. 1 and 2). Although this could suggest the potential for over-prediction of ZIKV cases for a national-based model, application of a U.S. model showed this to not be an issue. However, false prediction and timing of high ZIKV activity periods were apparent issues in the national model. That U.S. tweet counts responded less sensitively to ZIKV case counts, and that the U.S. model did not perform as well as the Florida model, makes sense given the higher spatial coverage of the entire U.S. relative to ZIKV hotspot regions (e.g. FL, CA, NY, and TX).

The keyword *mosquito* was also examined for its potential to serve as an early signal of locally acquired ZIKV, given that a rise in mosquitoes (the primary ZIKV vector) would expectedly lead to a rise in ZIKV. This keyword, however, provided no added benefit over use of the keyword *zika*. Rather, *zika* and *mosquito* tweets were tightly correlated throughout the entire year.

In general, the increase of ZIKV in the summer and subsequent decrease in the fall season can be explained by higher temperatures and humidity during summer months, which provides conditions ideal for mosquito breeding, as well as increased person travel. Additionally, pesticide spraying campaigns during the height of the outbreak, particularly in late summer, may have helped to control mosquito populations and prevent the spread of ZIKV. For instance, aerial spraying of the organophosphate pesticide Naled was conducted in Miami-Dade County, Florida, multiple times in September in order to combat ZIKV [66]. In addition to dropping temperatures in the fall, this was another likely contributor to the sharp decrease in ZIKV cases and related tweets during this season.

In terms of spatial distribution across the U.S., ZIKV case reports were highly correlated with population-adjusted *zika* tweets. States with the most ZIKV cases also had the highest *zika* tweet prevalence while states with the fewest cases had the lowest tweet prevalence. This suggests that in addition to temporal accuracy, Twitter data may be a useful tool for predicting disease prevalence spatially. Additionally, this reinforces the potential utility of using Twitter data for ZIKV disease surveillance at the national level.

More research is necessary to identify an appropriate national-level predictive model. Additionally, future modeling efforts should attempt to separate tweets indicating awareness from tweets indicating infection. This could be accomplished by conducting a detailed content analysis of *zika* tweets. For instance, researchers could assemble a list of keywords or phrases in order to filter out non-infection related *zika* tweets. Once validated, this approach would produce a new time-series dataset of *zika* tweet counts that could be used to calibrate a new predictive model of ZIKV case counts. This approach would enable us to understand the underlying relationships between tweets and case counts. Lastly, calibration of other state-wide models for comparison with our Florida model is a worthwhile area of future research in order to understand how the relationship between Twitter data and disease incidence might vary from state-to-state, and to better utilize such data for predictive purposes in other regions.

## Conclusions

*Zika* tweets exhibited a very similar temporal pattern as ZIKV case counts in the U.S. during the 2016 ZIKV epidemic. An auto-regression analysis using data from Florida showed *zika* tweets to be a significant predictor of ZIKV cases, with model evaluation demonstrating that weekly ZIKV case counts could be predicted 1 week in advance with reasonable accuracy. By comparison, a nationally calibrated model showed reduced, but still moderate predictive ability. Model performance was improved for both models with the inclusion of prior ZIKV case count data, as opposed to just Twitter data. This study suggests that Twitter data can serve to signal changes in disease activity during an outbreak period. Additionally, spatial mapping of ZIKV and *zika* tweets across the U.S. showed similar patterns. States with the most ZIKV cases had the highest *zika* tweet prevalence while states with the fewest cases had the lowest tweet prevalence, indicating that spatial ZIKV predictive modeling may be possible at the national level.

## Additional file

Additional file 1: **Table S1.** AIC values for Florida and U.S. candidate models and univariate models. **Table S2.** Results of zero mean test using Augmented Dickey-Fuller Unit Root Test. **Figure S1.** Weekly *zika* and *mosquito* tweets during the year 2016 in Florida. **Figure S2.** Results of the white noise test for the a) Florida and b) U.S. models. **Figure S3.** Auto-correlation function (ACF) plots for a) Florida and b) U.S. models. **Figure S4.** Distribution of residuals for the a) Florida and b) U.S. models. **Figure S5.** Scatter plot of residuals for the a) Florida and b) U.S. models. A very similar pattern in tweet frequency exists between the keywords *zika* and *mosquito*. Use of the keyword *mosquito* therefore provided no added benefit as a temporal indicator of ZIKV compared to the keyword *zika*. In terms of model selection, AIC values for various candidate models as well as univariate models for Florida and the U.S. are presented. Zero mean test results are also shown, suggesting that we can reject the null hypothesis that a unit root exists. Diagnostic results for the Florida and U.S. predictive models include an assessment of white noise, normality of residuals, heteroscedasticity, and partial auto-correlation (PACF). The white noise assessments indicate that more complex models were not necessary, while residuals plots assessing normality and heteroscedasticity of residuals suggest that residuals were not normally distributed for either model. In general, our models tended to over-predict at low case count values and under-predict at high case counts, thus suggesting a limitation of our models. PACF analyses support our choice as it relates to the number of lag terms used for the Florida model and the U.S. model. (DOCX 456 kb)

## Data Availability

The datasets used and/or analyzed during the current study are available from the corresponding author on reasonable request. Competing interests The authors declare that they have no competing interests.

## Abbreviations

AIC: Akaike Information Criterion
API: Application program interface
CDC: Centers for Disease Control and Prevention
RMSE: Root mean squared error
WHO: World Health Organization
ZIKV: Zika virus
