# Supplementary material for: Use of Twitter data to improve Zika virus surveillance in the United States during the 2016 epidemic
Source: BMC Public Health. 2019 Jun 14;19:761. doi: 10.1186/s12889-019-7103-8 (PMC6570872; doi:10.1186/s12889-019-7103-8)
Supplement: Supplementary file 1 — Table S1. AIC values for Florida and U.S. candidate models and univariate models. Table S2. Results of zero mean test using Augmented Dickey-Fuller Unit Root Test. Figure S1. Weekly zika and mosquito tweets during the year 2016 in Florida. Figure S2. Results of the white noise test for the a) Florida and b) U.S. models. Figure S3. Auto-correlation function (ACF) plots for a) Florida and b) U.S. models. Figure S4. Distribution of residuals for the a) Florida and b) U.S. models. Figure S5. Scatter plot of residuals for the a) Florida and b) U.S. models. A very similar pattern in tweet frequency exists between the keywords zika and mosquito. Use of the keyword mosquito therefore provided no added benefit as a temporal indicator of ZIKV compared to the keyword zika. In terms of model selection, AIC values for various candidate models as well as univariate models for Florida and the U.S. are presented. Zero mean test results are also shown, suggesting that we can reject the null hypothesis that a unit root exists. Diagnostic results for the Florida and U.S. predictive models include an assessment of white noise, normality of residuals, heteroscedasticity, and partial auto-correlation (PACF). The white noise assessments indicate that more complex models were not necessary, while residuals plots assessing normality and heteroscedasticity of residuals suggest that residuals were not normally distributed for either model. In general, our models tended to over-predict at low case count values and under-predict at high case counts, thus suggesting a limitation of our models. PACF analyses support our choice as it relates to the number of lag terms used for the Florida model and the U.S. model. (DOCX 456 kb) [file 12889_2019_7103_MOESM1_ESM.docx]

Additional file 1

**Table S1.** AIC values for Florida and U.S. candidate models and univariate models.

|  | Model Type | AIC |
| --- | --- | --- |
| Florida Models |  |  |
|  | AR(1,1) | 421.7 |
|  | AR(1,2) |  |
|  | AR(1,3) | 416.2 |
|  | AR(1,1) + Tweet _t-1_ | 410.5 |
|  | AR(1,2) + Tweet _t-1_ | 404.0 |
|  | AR(1,3) + Tweet _t-1_ | 396.4 |
|  | Tweet _t-1_ | 416.5 |
| U.S. Models |  |  |
|  | AR(1,1) | 558.2 |
|  | AR(1,2) | 551.2 |
|  | AR(1,1) + Tweet _t-1_ | 544.6 |
|  | AR(1,2) + Tweet _t-1_ | 539.2 |
|  | Tweet _t-1_ | 549.5 |

Table S1 shows AIC values for various candidate models as well as univariate models for Florida and the U.S. AIC values were used to determine the most appropriate predictive models and to ensure that models were not overfit. Only models that met specific criteria (see main manuscript) were considered candidate models and are shown below. The AICs of the chosen multivariate models were lower than other candidate models, and are underlined. Models with higher order AR terms [AR(1,4)-AR(1,6)] and higher tweet lags (2-6 week lags) were not significant and are not shown in Table 1. That models using tweet information had lower AICs than models that did not include such information suggests that the use of Twitter data produced improved models.

**Table S2.** Results of zero mean test using Augmented Dickey-Fuller Unit Root Test.

| Model | Type | Lags | Tau | p-value | |
| --- | --- | --- | --- | --- | --- |
| Florida Model | Zero Mean | 1 | -6.97 | <.0001 |  |
|  |  | 2 | -6.94 | <.0001 |  |
|  |  | 3 | -4.27 | <.0001 |  |
| U.S. Model | Zero Mean | 1 | -164.151 | <.0001 |  |
|  |  | 2 | -78.5982 | <.0001 |  |

Zero mean test results are presented in Table S2. The probability of the test statistic (tau) <0.05 indicates that we can reject the null hypothesis that a unit root exists. After first-order differencing, results show all p-values <0.0001. We can therefore conclude that no unit root exists and that the data series is stationary. Thus, further differencing (2^nd^ order, etc.) was not necessary.


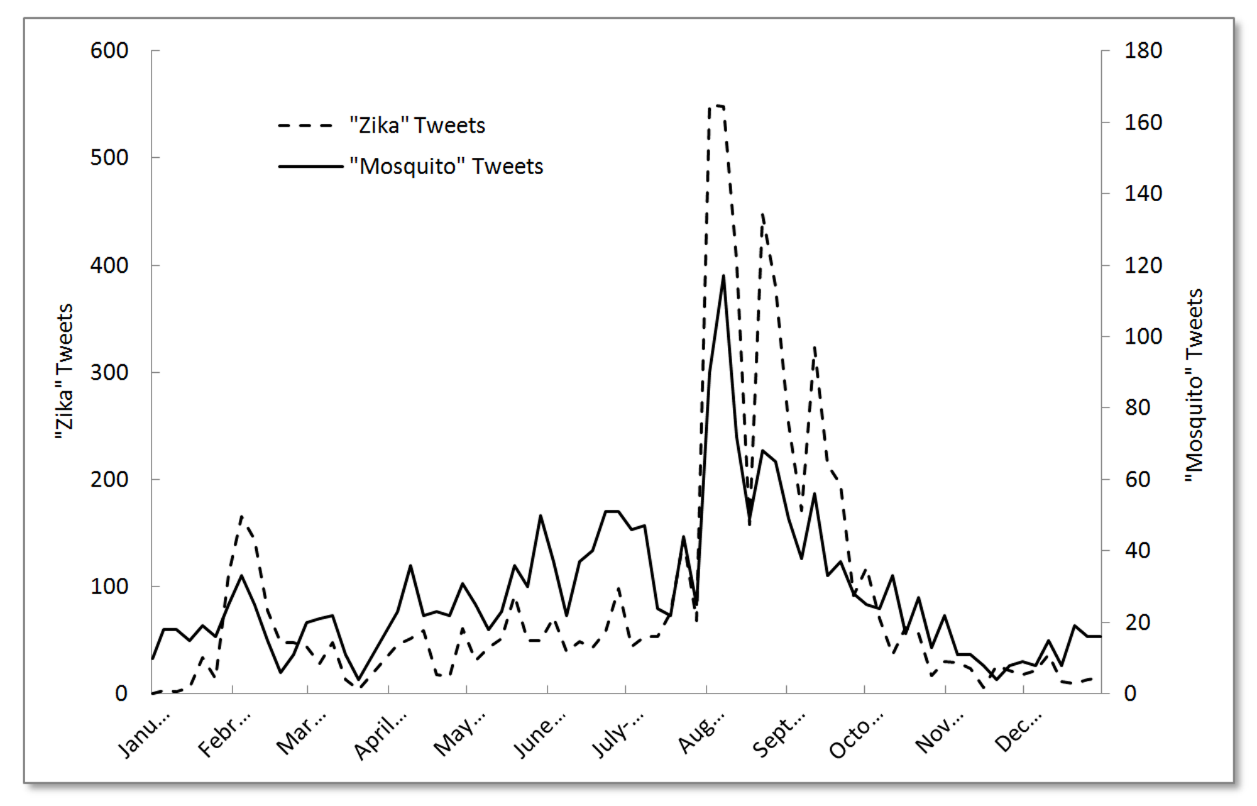


**Figure S1.** Weekly *zika* and *mosquito* tweets during the year 2016 in the United States.

Fig S1 is a time series plot of weekly *zika* tweets and *mosquito* tweets occurring in Florida in 2016. As shown, a very similar pattern in tweet frequency exists between these two keywords. Both keywords exhibit a distinct and temporally overlapping trimodal peak during the summer months, which gradually tapers off thereafter. Similarly, the sharp peak in *zika* tweets occurring in February is also apparent in *mosquito* tweets. Use of the keyword *mosquito* therefore provided no added benefit as a temporal indicator of ZIKV compared to the keyword *zika*. This is most apparent during the peak of the outbreak, when both keywords responded nearly identically over time.


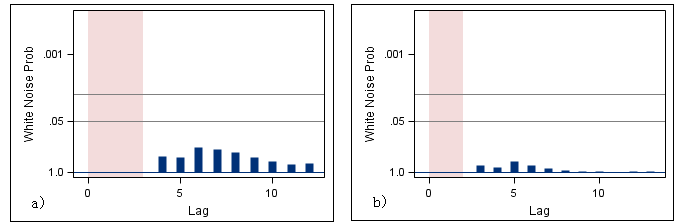


**Figure S2.** Results of the white noise test for the a) Florida and b) U.S. models.


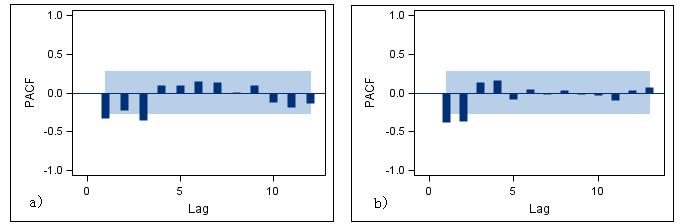


**Figure S3.** Auto-correlation function (ACF) plots for a) Florida and b) U.S. models.


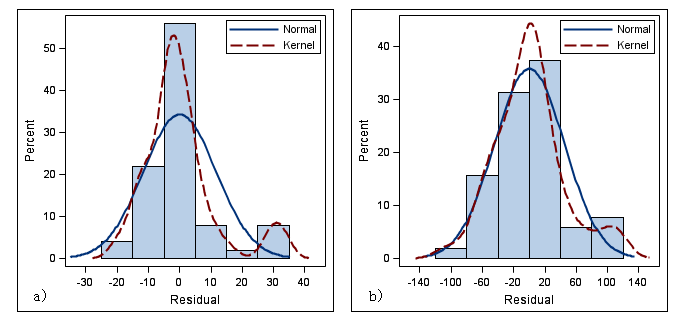


**Figure S4.** Distribution of residuals for the a) Florida and b) U.S. models.


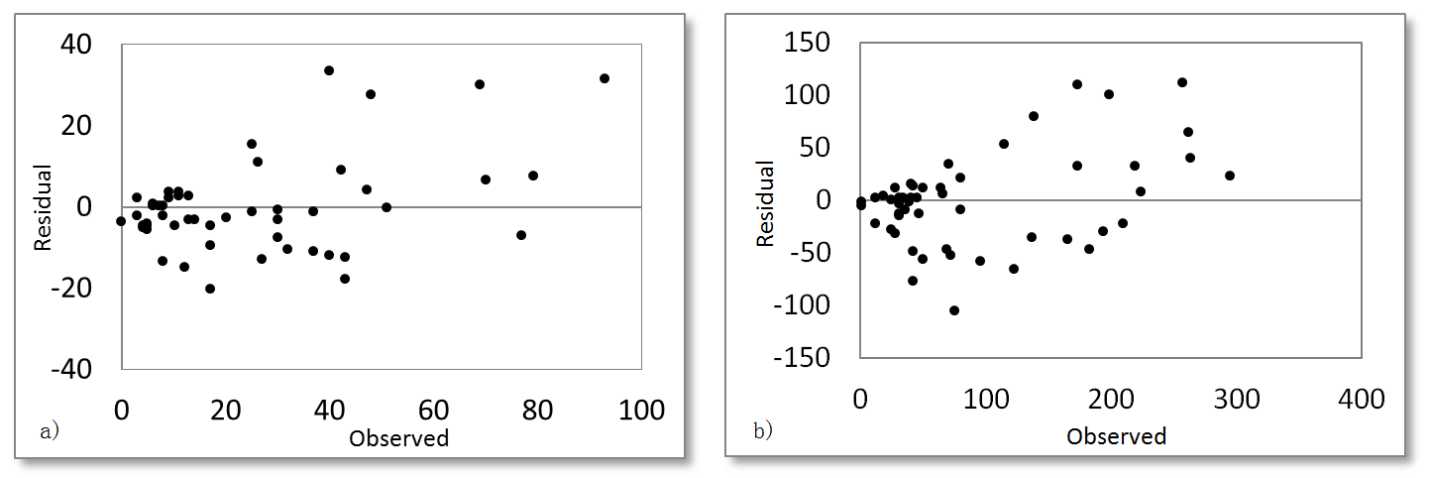


**Figure S5.** Scatter plot of residuals for the a) Florida and b) U.S. models.

Figs S2-S5 show diagnostic results for the Florida and U.S. predictive models, including the assessments of white noise, normality or residuals, heteroscedasticity, and partial auto-correlation (PACF). The white noise assessment tests whether the residuals are uncorrelated (white noise) or contain additional information that could be used by a more complex model. For both models, the plots in Fig S2 indicate that we cannot reject the hypothesis of no correlation of residuals; thus, the residual series can be assumed to be white noise and more complex models were not necessary.

Residuals plots assessing normality and heteroscedasticity of residuals are depicted in Figs S4 and S5, respectively. Residuals equaled the observed minus predicted values. Fig S4 shows that residuals are not normally distributed for either model. This is consistent with the Shapiro-Wilk test for normality, which yielded p-values less than 0.01, meaning we must reject the null hypothesis that the residuals are normally distributed over the data series. Fig S5 shows that our models tended to over-predict at low case count values and under-predict at high case counts. This represents a limitation of our models.

PACF plots are depicted in Fig S3. The PACF is the amount of remaining correlation between the outcome variable (weekly ZIKV cases) and a lag of itself that is not explained by correlations at all lower order lags. The PACF is useful for identifying appropriate lag terms for autoregressive models. In the case of the Florida model, the PACF drops sharply after three lags, while the same is true after two lags for the U.S. model. This supports our use of three lags terms (lag 1, lag 2, lag 3) for the Florida model and two lag terms (lag 1, lag 2) for the U.S. model.
